# Metallic Structures: Effective Agents to Fight Pathogenic Microorganisms

**DOI:** 10.3390/ijms23031165

**Published:** 2022-01-21

**Authors:** Diana Pereira, Tiago Soares Carreira, Nuno Alves, Ângela Sousa, Joana F. A. Valente

**Affiliations:** 1 CICS-UBI-Health Sciences Research Centre, Universidade da Beira Interior, Avenida Infante D. Henrique, 6200-506 Covilhã, Portugal; diana.carvalho.pereira1@gmail.com (D.P.); angela@fcsaude.ubi.pt (Â.S.); 2 CDRsp-IPL-Centre for Rapid and Sustainable Product Development, Polytechnic of Leiria, Marinha Grande, 2430-028 Leiria, Portugal; tiagosc112@gmail.com

**Keywords:** antimicrobial agents, bacteria, fungi, metals, virus

## Abstract

The current worldwide pandemic caused by coronavirus disease 2019 (COVID-19) had alerted the population to the risk that small microorganisms can create for humankind’s wellbeing and survival. All of us have been affected, directly or indirectly, by this situation, and scientists all over the world have been trying to find solutions to fight this virus by killing it or by stop/decrease its spread rate. Numerous kinds of microorganisms have been occasionally created panic in world history, and several solutions have been proposed to stop their spread. Among the most studied antimicrobial solutions, are metals (of different kinds and applied in different formats). In this regard, this review aims to present a recent and comprehensive demonstration of the state-of-the-art in the use of metals, as well as their mechanisms, to fight different pathogens, such as viruses, bacteria, and fungi.

## 1. Introduction

Over the years, multidrug-resistant pathogenic microorganisms have emerged due to the overuse and misuse of antibiotics (and other drugs) against different types of infections, and also because of pathogens’ innate ability to genetically acquire drug resistance [1]. Among the biggest enemies of antimicrobial drug treatments, is biofilm formation by microorganisms, since the extracellular polymeric substances from these biostructures work as an effective metabolic and physical barrier, developing antibiotic or antifungal resistance by making drug entrance difficult and consequently reducing effectiveness [2]. This poses a serious threat to public health as it becomes a challenge to treat the infections caused by these “superbugs” [3,4]. 

Most recently, the COVID-19 pandemic has emphasized how unprepared humanity is to fight threats associated with epidemic outbreaks of infectious diseases. Severe acute respiratory syndrome coronavirus 2 (SARS-CoV-2) is the latest in a long list of viral diseases that have emerged as severe threats to public health [5]. There have been made many attempts to develop medicines and vaccines to fight viruses; however, their ability for quick adaptation in their current host, and the ability to switch to a new host, are matters of great concern. 

An example of a possible therapeutic target against the infection induced by SARS-CoV-2 can be the main SARS-CoV-2 protease, which plays an important role in the virus lifecycle, through the application of cannabinoid receptor type 2, which is highly expressed in immune cells, inhibiting inflammatory processes [6,7]. Concerning cannabinoid use, Raj et al. (2021) found that these molecules are important to inhibit viral replication of SARS-CoV-2 in two ways: by binding to the main protease and blocking translation, and by acting as agonists for the cannabinoid receptor, therefore reducing the levels of pro-inflammatory cytokines [8]. Hence, there is still an urgent need to develop novel potential antiviral agents [9].

In this regard, metals have been used for centuries due to their exceptional ability to work as antimicrobial agents for different applications, such as in healthcare, food, and agriculture, among others. Moreover, the application of these kinds of materials as biocidal agents had grown so much that the global metal biocides market is projected to reach USD 3.49 billion by 2021 with a compound annual growth rate of 4.8% from 2016 to 2021 (https://www.marketsandmarkets.com/Market-Reports/metal-biocide-market-121653989.html, access on 4 November 2021).

To fight some of these pathogens, society has been using metals, such as mercury, silver (Ag), gold (Au), and iron (Fe), and, more recently, other types of metals, such as titanium (Ti) and zinc (Zn), among others. Each of these has various properties, potencies, and spectra of activity, and could be used in different formats that can range from surfaces and coatings to nano- or microparticles without losing their ability to kill microorganisms.

The main mechanisms of action of metals could be triggered by metal reduction potential and through metal donor atom selectivity and/or speciation. Additionally, another possible classification for these mechanisms can be based on where the biocidal metal acts: either in the cell membrane or in the intracellular region (more detailed information will be provided in the following section) [10].

Regarding the above mentioned, this review intends to present the latest advances in the field of the use of metals as biocidal agents, focusing also on mechanisms behind this effect. The presented approaches will address different structures that can be applied, such as surfaces or coatings, as well as nano- or microparticles. Antimicrobial solutions will be also presented, where metals are synergistically combined with drugs or polymers to increase the potency of the biocidal effect.

## 2. Mechanisms behind the Antimicrobial Effect

In general, metals represent a considerable portion of the periodic table, and their properties can correlate with reactivity in living cells. They are required for the correct functioning of many enzymes and are involved in nearly all biological processes, providing structural stability for proteins, cell membranes, and DNA; they are co-factors for enzymes; and redox potential to facilitate electron transport and catalysis [11,12].

Even though they are essential for many biological functions, metals can also be toxic and lethal when in excess. Among many things, they can increase intracellular reactive oxygen species (ROS) via Fenton chemistry, inducing oxidative stress; disrupting membrane function and cell growth processes; and damage DNA and negatively influencing enzyme activities and protein function [11,12,13].

The toxicity of metals is achieved through multiple mechanisms of action, such as reduction potential—which interferes with the electron transfer process, preventing cells from acquiring electrons that are essential to cellular functions, and donor atom selectivity—where a protein binds to the wrong metal cofactor and ends up with compromised folding or function [13].

Regarding the production of ROS, relevant mechanisms have been proposed through which this could be accomplished, such as the existence of other redox-active metals (such as Fe, copper (Cu), and nickel (Ni)), which can lead to Fenton chemistry, the destruction of Fe–sulfur (S) protein clusters, resulting in the release of additional Fe into the cytoplasm, which is Fenton-active and able to produce ROS in excess; and, last but not least, the oxidation of thiols within cells, which leads to the production of S radicals, forming protein disulfides and depleting glutathione reserves, leaving protein targets susceptible to attack by metals or ROS [11].

Furthermore, some metals apply their toxicity outside the microorganism barrier, by simply binding to the membrane, while others can bind to ligands of low molecular mass that present functional groups, such as phosphate, amino acids, or peptides, and be co-transported into the cell. Once inside a cell, they can cause a loss of enzyme activity, protein dysfunction, genotoxicity, and interfere with the assimilation of nutrients, which leads to growth arrest via starvation. Additionally, some metals, such as Ag, can interfere with the electron transport chain by interfering with sodium ion (Na^+^) [11].

Despite the above-mentioned points, the different metabolisms of microorganisms in combination with the multiple processes evolved in terms of their growth inhibition/death, it is difficult to fully understand metal-derived toxicity. Nevertheless, electrostatic interactions, established between the metal ions (positively charged) and the components of the bacterial cell wall (negatively charged), are indicated as the main effect [14].

## 3. Metallic Particles—Nano- and Microstructures

### 3.1. Nanoparticles

Nanomaterials are reported to have dimensions between 1–100 nm. Due to their small size, nanoparticles (NPs) can be used for biosensing, drug delivery, bioimaging, catalysis, nanomanufacturing, lubrication, electronics, in the textile industry, and for water treatment. They can also cooperate with biomolecules inside cells or on the cell surface, carrying drugs or genetic materials, such as DNA, RNA, or small interfering RNA (siRNA), into target cells or tissues for gene expression [15,16].

The use of NPs as antimicrobial agents could overcome mechanisms of bacterial resistance due to their microbicidal nature, which results from direct contact with the bacterial cell wall, without the need to penetrate the cell [16]. Therefore, there is less chance to develop antibacterial resistance to NPs when compared to antibiotics, allowing NPs the potential to be used for antimicrobial theragnostics in medicine [17]. Metal NPs (M-NPs) have been shown to have in vitro antimicrobial activity against a wide range of organisms, including Gram-positive and Gram-negative bacteria, mycobacteria, fungi, and viruses [15,17].

These kinds of NPs can be produced using two different methods: top–down and bottom–up. A top–down method is a destructive approach that starts from a larger molecule that is decomposed into smaller units, which are then converted into suitable NPs. The bottom–up approach is performed in reverse, with the NPs being formed from relatively simpler substances [18,19]. Table 1 describes different methods applied for M-NPs production.

These metallic nanostructures have several applications in numerous fields, such as in diagnostics, drug delivery, antimicrobial activity, and the treatment of several diseases [39]. M-NPs are widely recognized to have antimicrobial properties and are already widespread as antimicrobial agents with several products for antimicrobial purposes having been approved by regulatory agencies and commercialized. Some of these NPs have been used in combination with certain antibiotics to help overcome resistant bacteria, enhancing the antibiotic effect [40]. In addition, nanomaterials can be moulded to incorporate conventional antiviral properties with modifications that are exclusive to nanosystems, such as small and controllable sizes that can represent a solution for the treatment of viral infections [41,42].

The main components of the bacterial cell wall are peptidoglycans linked to teichoic acid, glycolipids, and phospholipids, which provide a negative charge to the cell wall. The interaction between the positively charged metal ions and the cell wall is enabled by this negative charge, which leads to a loss of membrane integrity. This damaged membrane can cause a leakage of cytoplasmatic content, such as minerals and genetic material, with a consequent rupturing of the cell, leading to cell death. Furthermore, if M-NPs enter the bacterial cell, they poison the microbial cells by generating ROS (which mediate cellular damage) or through metal-catalyzed oxidation reaction that could lead to disfunction of proteins or DNA and interfere with the assimilation of nutrients by the cell [43].

Nguyen and co-workers studied the antimicrobial mechanisms of magnesium oxide MgO-NPs, and SEM images of Gram-negative bacteria, such as *Escherichia coli* (*E. coli*), showed membrane damage when they were seeded with high doses of MgO-NPs. These Gram-negative bacteria present a thin layer of peptidoglycan between the phospholipid layers so the MgO-NPs can easily pass through the cell wall and bind to the membrane, causing shape distortion and cell death. On the contrary, Gram-positive bacteria present a thick surface layer made of peptidoglycan that retains the MgO-NPs. This characteristic can inhibit cell growth and prevent cell adhesion to surfaces [44]. Shankar and Rhim also reported a difference between Gram-positive and Gram-negative bacteria, regarding antimicrobial activity when Ag-NPs were used. Similarly, Ag-NPs also experience more difficulty in penetrating the peptidoglycan layer (20–80 nm) in Gram-positive bacteria, in contrast to what happens to the outer membrane of Gram-negative bacteria (7–8 nm) [45].

In particular, with Ag-NPs, it has been proposed that the release of positively charged silver ions (Ag^+^) may interact with the negatively charged proteins or nucleic acids, damaging the structure and deforming the bacterial cell walls and membranes. This phenomenon will disrupt metabolic processes, leading to eventual cell death. It has also been suggested that ROS derived from the NP surface causes membrane damage by increasing membrane permeability, which will lead to cell death [45,46,47]. Chatterjee and collaborators (2015) also reported that, after treatment with AG-NPs, the cellular DNA of *E. coli* and *Staphylococcus aureus* (*S. aureus*) showed condensation, and *E. coli* cells were more susceptible to the treatment compared to *S. aureus*, probably due to the difference in structure, which has been previously described in the literature [46].

Iron NPs could be applied per se as antimicrobial agents or can act as hyperthermia agents to treat bacterial infections (this kind of microorganism shows a higher temperature susceptibility than healthy human host cells) [48]. When placed under an alternating magnetic field with a high frequency and amplitude, these particles will absorb electromagnetic radiation and subsequently convert the magnetic energy into localized heat, leading to bacteria death [49]. The study by Kim et al. (2013) can be cited as a good example of an effective application of Fe-magnetic NPs combined with hyperthermia to provide antimicrobial efficacy in a mouse infection model caused by *S. aureus* [50].

Similar to what happens with bacteria, antifungal mechanisms provided by M-NPs can be associated with a disturbance of fungal cell membrane integrity, and with the generation of ROS, which enhances the membrane disintegration process [51]. With metal toxicity comes a depletion of glutathione and a loss of antioxidant response to oxidative stress [52,53]. Table 2 provides different examples of experiments regarding the antimicrobial action of M-NPs against several pathogens.

**Figure 1:**
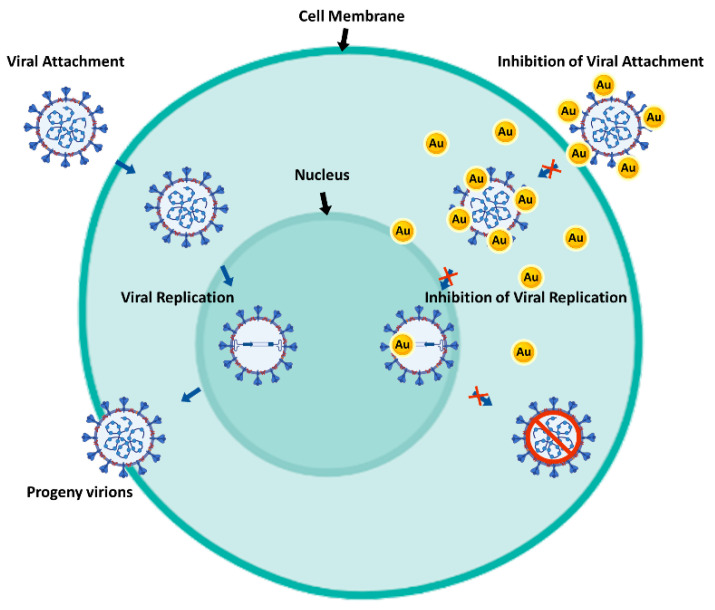
Representative mechanism of the antiviral properties of Au-NPs. On the left, a normal mechanism by which a virus infects a host cell (in this case, a eukaryotic cell) can be seen: firstly, viral attachment to the cell membrane occurs, followed by penetration into the cytoplasm; then the virus proceeds to use the cellular mechanisms to replicate its genetical material, generating virions to continue the infectious cycle. On the right, is shown a mechanism of inhibition by which Au-NPs can intervene, either by attaching themselves to the virus, blocking its attachment to the cell and consequent entry in the cell and ultimately interfering with the mechanisms of viral replication inside the cell (adapted from [88]).

Antiviral mechanisms of M-NPs can occur either inside or outside host cells, as can be seen in Figure 1. These mechanisms include a competition between NPs and the virus for host cell-binding sites, to prevent viral attachment, and blocking virus–host binding or penetration. This kind of mechanism could be observed in recent research work performed by Jeremiah and co-workers, where Ag-NPs effectively inhibited extracellular SARS-CoV-2 by protecting target cells from infection. Another mechanism consists of inactivating virus particles before cellular entry, interacting with the viral genome, or binding to the viral particles, which can also be considered as potential mechanisms of action [84]. Furthermore, the intracellular compartment of an infected cell is abundant in the virally encoded and host cellular factors required for viral replication and production of virions. Hence, another antiviral mechanism of action is the interaction of M-NPs with these replication factors [9,86]. Overall, different NPs present different antiviral mechanisms of action, for example Au-NPs (similarly to Ag-NPs) include the blocking of glycoprotein envelope (gp120) attachment to inhibit viral entry or replication (as can be seen in Figure 1); Cu-NPs can destroy the viral genome and disrupt the capsid, Zn-NPs interfere with viral DNA polymerase activity, resulting in viral replication inhibition, and iron (Fe) NPs (Fe-NPs) bind to the virus to inhibit its binding to cells [9,87].

Overall, there are many studies in the literature stating that the complete mechanism of action underlining the antimicrobial activity and efficacy of M-NPs is not fully clear or understood; however, among the parameters that influence the biocidal effects of metals are the size, shape, and metal ions involved [87]. With bacteriathis influence diverges between Gram-negative and Gram-positive cells, where M-NPs have more difficulty in passing through the thick layer of peptidoglycan in the Gram-positive cell wall, but, once inside the cell, the principles are the same. The antifungal mechanisms of M-NPs are similar to the antibacterial ones, which are the rupture of the cell membrane by oxidative stress and leakage of DNA and proteins. Regarding the antiviral mechanisms of M-NPs, they compete with the virus for cell-binding sites, therefore, inhibiting virus attachment to the cell, or preventing viral replication inside the cell.

### 3.2. Microparticles

Microparticles (MPs) are spherical particles that range in size from 0.1 to 1000 μm and can be synthesized from natural and synthetic materials, such as polymers, glass, and ceramics. Similar to what happens with NPs, their small size gives them an advantage over larger particles (macroscale) and presents many desirable properties for a variety of different applications, such as drug delivery [89,90].

In general, MPs are very similar to NPs in terms of their synthesis methods and applications, except for their size, which is bigger. Similar to NPs, MPs are commonly used to promote antimicrobial effects in different industries, and can be used as a drug delivery system due to their ability to protect from degradation bioactive drugs, proteins, and small molecules; they are also capable of controlling the release rate of encapsulated drugs over an extended period, up to months, and also, they are easily processed [89,91].

Some studies have been conducted regarding the use of MPs against different pathogens. For instance, Sophee and co-workers synthesized titanium oxide (TiO_2_) and ZnO MPs using the sol–gel method to prevent water-borne microbial infections. From the obtained results, it was possible to enhance the antibacterial activity against Gram-negative bacteria (*E. coli* and *K. pneumoniae*) and Gram-positive bacteria (*Streptococcus pyogenes*) [92]. More recently, Steckiewicz and coworkers studied spherical Ag-orthophosphate-MPs, produced by chemical precipitation, against Gram-positive bacteria (*S. aureus*) and fungi/yeast (*C. albicans* and *A. niger*). The application of these MPs led to increased levels of ROS and first-line defense antioxidants, such as superoxide dismutase and glutathione peroxidase, which could lead to biomedical applications in implant-related infections [93].

Due to their difference in size, MPs take more time to dissolve than NPs; therefore, they release fewer metal ions in the same period of time. As such, NPs usually present a better alternative with stronger and better antimicrobial effects [10,93].

## 4. Antimicrobial Surfaces and Coatings

As mentioned above, pathogen resistance has prompted the development of antimicrobial surfaces. A variety of inorganic materials (namely metals, metalloids, metal alloys, metal ions and oxides) can be used to grant surfaces effective antimicrobial properties [1,94]. In fact, not only can they be used as alternative antimicrobial materials, but they are also advantageous as they present more stable antimicrobial action and are a “green” approach (eco-friendly) when compared to polymers and chemical surfaces [1].

To fight these multi-resistant infections, the propagation of pathogens needs to be stopped. For this matter, several researchers have developed different antimicrobial surfaces with biocidal and/or anti-adhesive properties [95,96].

Amongst the wide range of metals, Cu and Ag are the two most commonly used, with proven antimicrobial properties, such as the ability to effectively inhibit and kill different bacterial and viral strains upon direct contact, either as coatings or as the surface material itself [97,98]. This happens due to the release of metallic ions that become toxic to microorganisms at high concentrations, leading to membrane rupture and, therefore, cell death. Between both metals, Cu proves to be advantageous compared to Ag, as it exhibits the best antimicrobial results and is also cheaper to acquire. For this reason, a large number of Cu-containing surfaces have been studied and been registered to have antimicrobial activity against several microorganisms [1,94,96,98]. Several applications for Cu surfaces, as well as for other kinds of metallic surfaces with proven results on the different microorganism reductions are described below:

Healthcare applications

Metals have been used for healthcare applications to prevent the spread and promote dead of pathogens over human history. In this context, several researchers have been studying the effect of metallic surfaces when in contact with different pathogens. For example, Noyce and co-workers compared the antiviral effect of Cu and stainless-steel surfaces by inoculating the Influenza A virus in sterile coupons of both metals for periods between 1 and 24 h [99]. After, the number of viral particles that remained infectious after the incubation process was determined, resulting in a 75% reduction of active virus after 24 h with stainless steel. Cu managed the same reduction (75%) in just 1 h, further reducing the number of infectious viruses to under 0.1% after 6 h of initial inoculation, emphasizing Cu’s great antimicrobial potential [90]. In another study, Warnes and collaborators investigated the antiviral activity of a wide range of Cu-containing alloys, including Cu-Nis and brasses, as well as Cu, Ni, and Zn in their pure metal forms (as controls) against human coronavirus 229E (HuCoV-229E) [100]. To investigate the antiviral effects of these metals, they spread HuCoV-229E over 1 cm^2^ coupons of the various test surfaces before incubating at room temperature for different periods of time. Their main results indicated that plain Cu, brasses containing over 70% Cu, and Cu-Nis with over 90% Cu, displayed the best antiviral effects against HuCoV-229E, as some of the alloys completely inactivated 100% of the inoculated virus after only 20 min of exposure [100]. Coincidently, some of the alloys used against HuCoV-229E were also used against *E. coli* O157 by Wilks and co-workers under similar conditions (using metal coupons) and the results obtained against these Gram-negative bacteria were similar to those observed against the virus [101]. C21000 (95% Cu) and C23000 (85% Cu) alloys, which were used in both cases, showed some of the best antiviral and antibacterial results in their respective works. C21000 managed to completely eradicate HuCoV-229E in 20 min, while it took a little longer to kill 100% of *E. coli* O157 (90 min). As for C23000, total microbial eradication was achieved after 60 min for both HuCoV-229E and *E. coli* O157, displaying the best antibacterial activity in the corresponding study [99,101]. These results indicate that brasses containing over 85% Cu could be a viable option for the fabrication of antimicrobial surfaces for general use.

The antimicrobial activity of Cu is effective against several pathogenic microorganisms, namely bacteria and viruses, as evidenced in this review paper. However, Cu’s inhibitory effect against pathogens also extends to fungi. Ballo and collaborators described the fungicidal activity of Cu-coated surfaces against azole-resistant *C. albicans* and *C. glabrata* [96]. In this study, Cu-sputtered polyester surfaces (Cu-PES) and uncoated PES (control) were used and inoculated with the above-mentioned fungal strains, for different periods (15, 30, or 60 min) at room temperature and under either dark or actinic light. The authors found that Cu-PES displayed fungicidal activity against both *C. albicans* and *C. glabrata* under dark and actinic light conditions within 60 min [96].

Recently, the SARS-CoV-2 pandemic hit the globe, becoming a main focus for science to determine how to prevent its spread. Concerning this, several researchers worldwide started to work hard on this problem. Among them, Hutasoit and collaborators explored the viricidal activity of cold-sprayed Cu-coated surfaces against SARS-CoV-2 in an attempt to slow the dissemination of this virus (as well as other viruses) [102]. The cold-spray coating method used in their work is highly advantageous because it allows a very rapid coating “treatment” of commonly touched steel surfaces, which is faster and cheaper than replacing every single one of these surfaces that are touched by the public. This cold-spray technique consists of propulsion of metal particles at supersonic speeds onto surfaces due to a highly pressurized carrier gas, forming a thin dense metal layer. For antiviral testing, a push plate was subjected to an annealing treatment (Cu A) while another was tested without further treatments (Cu N). After preliminary tests, they proceeded to study the viricidal effect of Cu coating by placing SARS-CoV-2 on small squares of Cu (Cu N), stainless steel and activated Cu (Cu A) at room temperature at different times (1, 10, 30, 120 and 300 min). Their main results showed a 96% viral inactivation after 2 h of exposure to the Cu N coating and a 92% inactivation on the Cu A coating after the same exposure time. After 5 h of contact, the viral inactivation efficiency increased to 99.2% and 97.9%, for the respective coatings [102].

Other researchers have also studied surfaces with physical and/or chemical modifications. As an example, Selvamani and collaborators demonstrated the antimicrobial properties of a laser textured Cu (LT-Cu) surface and its efficiency against pathogenic bacteria, such as methicillin-resistant *S. aureus* (MRSA), *P. aeruginosa*, *S. aureus*, and *E. coli*, at different concentrations [1]. According to their findings, the LT-Cu process enhanced the bactericidal properties of the surfaces, achieving total eradication of *E. coli* after 120 min of direct exposure. Moreover, they studied the bacterial killing potential of simple (not modified) Cu surfaces and LT-Cu surfaces by dipping both surfaces in PBS (phosphate-buffered saline) with two multidrug-resistant bacterial strains (MRSA and *P. aeruginosa*) and found that LT-Cu surfaces completely eradicated *P. aeruginosa* and MRSA after 40 and 90 min of exposure, respectively. The pristine Cu surfaces also killed 100% of both strains but at a slower pace. These authors found that, when higher concentrations of *S. aureus* and *E. coli* were applied, only LT-Cu surfaces were able to eradicate them (at 120 and 60 min of exposure). Moreover, the LT-Cu process used by these researchers produced micro and mesoporous structures, which in turn granted anti-adhesive properties to the surfaces that might have helped to improve their antibacterial activity [1].

Taking the above-mentioned into account, and to create the “ultimate” antimicrobial surface, a combination of anti-adhesive surfaces and biocidal agents was proposed by Ellinas and co-workers, as well as Kefallinou and co-workers, due to the limitations of both types of surfaces when used separately (e.g., biofilm formation reduces the biocidal activity of surfaces without anti-adhesive properties, while in anti-adhesive surfaces with no biocidal agents the anti-adhesive properties are lost over time, as the few attached microorganisms grow and form biofilms) [4,95]. These works consist in the development and study of the antimicrobial activity of micro-nanotextured superhydrophobic poly(methyl methacrylate) (PMMA) surfaces complemented with sputtered Cu and Ag coatings, which they referred to as “hybrid” surfaces. Overall, amongst several tests and comparisons between surfaces, they concluded that Cu was the best bactericidal agent and the Cu-coated superhydrophobic PMMA surface achieved the best antibacterial activity, as it demonstrated antimicrobial action against *Synechococcus* spp. PCC7942 (at one of the highest concentrations ever reported) [4,95].

Food industry and other applications

Metal surfaces and coatings have also been widely applied in the food industry as an attempt to fight cross-contamination (which commonly occurs between food, surfaces, and equipment). Concerning this, Akhidime and co-workers studied the leaching potential of Ag, Ti, Cu, Fe, molybdenum, and Zn into media and its effect against *S. aureus*, *E. coli* and *L. monocytogenes*, with silicon substrates as base surfaces (control) [97]. One of their antibacterial studies was conducted by submerging metal-coated surfaces into bacterial suspensions and registering the number of viable cells over time via plate counts. Among the main results, it was possible to observe that the biggest leaching behavior was obtained for Cu followed by Zn. Surprisingly, Ag demonstrated one of the lowest leaching behaviors. However, despite Ag’s leaching potential being 350 times lower than Cu, Ag displayed one of the best antimicrobial effects. Cu’s antibacterial potential was the greatest, killing 99% of the three bacterial species after just one hour of incubation. In the same period, Ag managed to kill 46%, 99%, and 34% of *S. aureus*, *E. coli*, and *L. monocytogenes*, respectively. Overall, Cu was the coating with the best antimicrobial activity, followed by Ag as the second-best coating against the three pathogenic bacterial species tested (*E. coli*, *S. aureus*, and *L. monocytogenes*) [97].

Another approach to the treatment of fungal infections using metals was presented by Bastos and co-workers with a study of the antimicrobial effect of gallium (gallium nitrate III, [Ga(NO_3_)_3_]) against different fungal pathogens, namely azole-resistant *A. fumigatus* and different species of *Candida spp* [103]. They performed a fungal killing assay by testing different concentrations of Ga(NO_3_)_3_ against various strains. For this, inoculated microplates were incubated at 37 °C for 2, 8, 16, 24, and 48 h in different media, and samples of each time were taken to measure cell viability. Based on their results, the researchers concluded that the antifungal activity of gallium is dependent on the Fe concentration in the medium, as gallium’s MIC increased (or no antifungal effect was obtained) when Fe-containing media were used [103].

Despite all the above-mentioned information, metal surfaces and coatings alone are not the sole options for the development of antimicrobial surfaces. Wettability is a property of materials that have been extensively studied to accomplish antimicrobial surfaces, through either super hydrophobicity or super hydrophilicity, due to their ability to reduce microbial adhesion [1,103]. When pathogenic adhesion is reduced, further growth and dissemination are hindered, which passively prevents infections [4,95,103]. Moreover, roughness can also cause a surface to become superhydrophobic, due to the entrapment of air between roughened structures when a liquid comes in contact with the solid surface. This entrapped air has an anti-adhesive effect that hinders microbial adhesion, as it reduces the area available for microorganisms to attach, therefore minimizing adhesion to surfaces with moderate wettability, such as food or food packaging [104].

Table 3 presents a summary of several studies using different approaches for the development of antimicrobial surfaces.

## 5. Synergic Combination with Drugs

**Figure 2:**
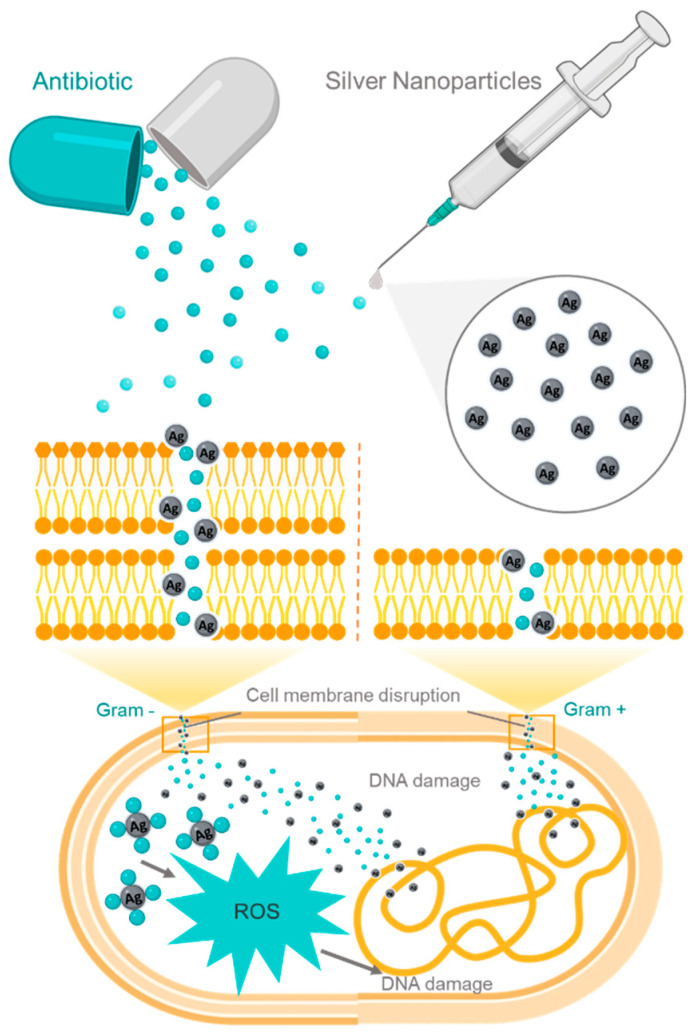
Representative illustration of the interaction between antibiotics and Ag-NPs and their combined action inside both Gram-negative (**left**) and Gram-positive (**right**) bacteria. Although there is a thicker path for Ag-NPs and antibiotics to travel through with the Gram-negative bacteria membrane, once, inside the cell, the mechanisms are the same as for Gram-positive bacteria: accumulation of particles inside the cell; production of ROS; and ultimately cell death by damaging the bacterial DNA (adapted from [111]).

Metallic particles could work with standard drugs to increase their antimicrobial activity. Moreover, the majority of practical trials in the literature regarding the synergistic effect between metals and drugs use Ag-NPs combined with antibiotics. Figure 2 presents an example of the combined action of antibiotics with Ag-NPs. M-NPs can disrupt the bacterial membrane of both Gram-negative (left) and Gram-positive (right) bacteria, enabling the entrance of antibiotics into the cell and leading to DNA damage. Moreover, the combined action of antibiotics with M-NPs induces oxidative stress and creates ROS; consequently, it also damages the bacterial DNA (as noted above) [106,107,108,109,110,111].

This effect, increased by the dual antimicrobial vehicles produced using M-NPs and different drugs, has already been demonstrated by several authors, as presented in Table 4. Some authors have studied the synergy of M-NPs in combination with antibiotics, such as amoxicillin and polymyxin B, and found higher bactericidal activity by binding Ag-NPs with these antibiotics [112,113]. For example, Abo-Shama and co-workers detected the antimicrobial activity of Ag-NPs and ZnO-NPs alone and combined with a range of antibiotics [114]. From nearly all the different antibiotic classes against Gram-positive and Gram-negative bacteria (*S. aureus*, *E. coli*, *Salmonella* spp. and *C. albicans*), their findings showed that the combination of Ag-NPs with some of the tested antibiotics presented a better synergistic effect than ZnO-NPs with the same antibiotics [114]. In addition, Vazquez-Muñoz and collaborators evaluated the antimicrobial activity of Ag-NPs combined with several antibiotics, such as chloramphenicol, kanamycin, ampicillin, aztreonam, and biapenem in both Gram-positive and Gram-negative bacteria [106]. They determined that Ag-NPs change the cell membrane integrity and increase cell permeability, enabling antibiotics intracellular access, which will enhance the efficiency of the antibiotics on their intracellular targets [106].

In another study, Pereira and co-workers produced chitosan microspheres and evaluated their efficiency in the adsorption of Ag ions for the formation of NPs [115]. Then, they evaluated the bactericidal activity and potential as a system for the release of ibuprofen. They found that the concentration of ibuprofen retained in chitosan microspheres was higher than in Ag-NPs microspheres, which indicates that microspheres with Ag-NPs released more drug (77%), exhibiting bactericidal characteristics and a greater drug release capacity than the material without Ag-NPs [115].

Although the majority of the research performed until now has focused on the synergistic interaction between Ag-NPs and antibiotics, other (fewer) experiments have been performed on other types of M-NPs conjugated with different drugs, such as antifungal or antiviral medicines, proving the versatility of M-NPs for a wide range of biomedical applications.

## 6. Conclusions and Future Perspectives 

Metals are very interesting and promising vehicles to promote antimicrobial effects. Through this review, it is possible to access a compilation of several studies, showing that different metals can be used in different types of “structures” to achieve/increase biocidal behavior. For example, metal particles have been shown as efficient approaches to promote antimicrobial effects. Although they can be applied as micro- or nanostructures, the nano size is usually desirable to be applied in biomedical fields. Some works have reported that NPs, particularly Ag-NPs, can present good synergistic relations with antibiotics, resulting in an enhanced antibacterial effect on resistant strains of bacteria. Some authors have even proposed that this relation and the type of response could be transposed to studies with viruses, although there is a need for additional research in this field. Although several advantages regarding the use of M-NPs have been highlighted, there is still a need to improve many aspects, such as their production methodologies, which should be mostly achieved through biological synthesis methods, trying to use few chemicals, as well as generating lesser amounts of waste.

Regarding metallic surfaces and coatings, according to the examples presented here, Cu has proven to be the metal with the best biocidal properties against several microorganisms. In addition, anti-wetting properties were revealed to be important to improve the antimicrobial activity of metal surfaces or metal-coated surfaces. Overall, a variety of approaches for the development of these self-disinfecting surfaces were able to accomplish just that—create surfaces that kill pathogenic microorganisms, such as bacteria, viruses, and fungi upon direct contact. This is true even against the most recent threat to humanity, the SARS-CoV-2 (COVID-19) pandemic.

As for future projects, the study of dual functionality (involving the combination of biocidal agents with anti-adhesive surfaces) might be the key to the development of superefficient multifunctional and multimicrobial killing agents.

## Figures and Tables

**Table 1:** Different methods for M-NPs synthesis.

	Method	Graphical Representation	Short Description	Reference
Top-Down synthesis	Mechanical Milling	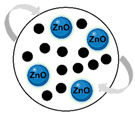	This approach includes the disintegration of particle aggregates, particle shape, and particle surface.	[20,21,22,23]
	Chemical Etching	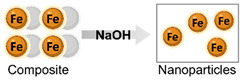	Uses a strong acid or a corrosive liquid to cut a metal surface and create a design in the metal.	[24,25]
	Sputtering	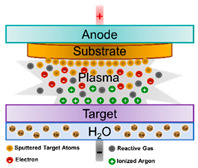	There is a particular sputtering technique called magnetron sputtering, which consists of the delivery of a high voltage across a low-pressure gas (normally argon) to create a plasma of high energy composed of electrons and gas ions, which will strike a target containing the desired coating material.	[26,27]
	Laser Ablation	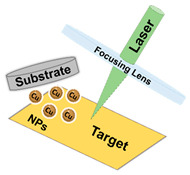	Is based on the production of micropatterns through the ablation (removal) of fractions of a substrate through the action of a focused pulsed laser beam.	[20,28]
	Electro Explosion	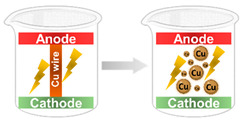	Single-step process in which a delicate wire of a conductive metal is exploded by an electric discharge that is caused by a high-power DC source. This electronic discharge creates a massive temperature that vaporizes the thin wire, turning it into gas atoms, which in their turn are chilled and, finally, the NPs are synthesized.	[29,30]
Bottom-up synthesis	Chemical Deposition	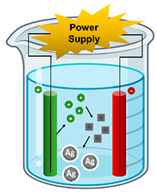	In particular, electrochemical deposition or electrochemical precipitation involves the passage of an electric current between an anode (sacrificial) and a cathode localized in an electrolyte. The anode is oxidized into metal ions and these are then reduced to metal by the cathode with the help of stabilizers.	[31,32]
	Spinning	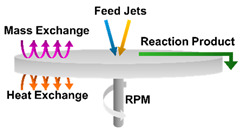	This method involves the use of a spinning disc reactor. The disc spins at different speeds and the spinning causes the fusion and precipitation of atoms, which are then collected.	[5,33]
	Sol–Gel	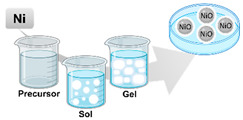	A wet-chemical process, with sol being a colloidal solution of solids suspended in a liquid phase that serves as a metal precursor and is then dispersed into the gel, a host liquid leading to the formation of a solid macromolecule submerged in the solvent. After, there is a separate phase where the gel is dried and dehydrated to recover the NPs.	[20,24,33,34,35]
	Biosynthesis	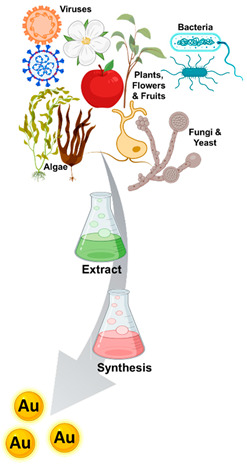	Metalsynthesis is bio-mediated by microbes or through biosynthesis. Biological synthesis of nanomaterials is the best alternative being cost-effective, environmentally friendly, advantageous and does not comprise any input of toxic chemicals. Between the candidates to biosynthesize M-NPs are bacteria, fungi and yeast; algae; plants, flowers and fruits; and viruses.	[18,19,23,36,37,38]

**Table 2:** Summary of some experiments regarding the antimicrobial action of M-NPs against different pathogens.

NPs	Microbes	Synthesis Method	Applications	Main Results	Reference
Zn	Gram-positive bacteria: *Streptococcus pneumonia, S. aureus*, and *Bacillus subtilis* Gram-negative bacteria: *Salmonella typhimurium, Pseudomonas aeruginosa*, and *E. coli O157:H7* Fungi and Yeast: *Candida albicans, Candida glabrata, Candida guilliermondii*, and *Candida krusei*	Green synthesis by *Ziziphora clinopodioides* extract	Cutaneous wound healing; Medical, biomedical and pharmaceutical research	Antibacterial, antifungal and antioxidant effects, without any cytotoxicity. Wound healing with increased levels of wound contracture, vessel, hydroxyl proline, hexosamine, hexuronic acid and fibrocytes/fibroblast rate. Growth inhibition concentrations were 1 mg/mL for *C. guilliermondii* and *C. krusei*, 2 mg/mL for *P. aeruginosa, S. aureus, S. pneumonia, B. subtilis, C. albicans* and *C. glabrata*, and 4 mg/mL for *E. coli* and *S. typhimurium.*	[54]
	Gram-positive bacteria: *S.aureus, Mycobacterium tuberculosis*	Extracellular by *Pseudomonas hibiscicola*	Drug-resistant strains hospital-acquired infections, treatment and prevention therapy.	Antibacterial potential. Synergism with gentamicin against MRSA. The minimum inhibitory concentrations (MIC) were 2.5 mg/mL for *S. aureus* and 1.25 mg/mL for *M. tuberculosis.*	[55]
	Virus: Hepatitis A	Hesperidin mediated synthesis	Drug development	Hesperidin-mediated ZnO NPs exhibit better antiviral activity than hesperidin alone. Hesperidin and ZnO NPs showed antiviral activity against Hepatitis A virus (HAV) with EC_50s_ equal to 72.4 and 176.3 μg/mL.	[56]
ZnO	Fungi and Yeast: *C. Albicans*	Green synthesis by *Prosopis farcta* aqueous extract	Industrial and medicinal	Antifungal effects and reduction of cell viability in cancer cells by increasing the ZnO-NPs concentration. The antifungal activity of ZnO-NPs against *C. Albicans* has shown that the MIC and minimum fungicidal concentration (MFC) were 128 and 256 μg/mL, respectively.	[57]
	Virus: H1N1 Influenza	-	Biomedical	Antiviral activity and reduction of cell cytotoxicity in MDCK-SIAT1 cells with a maximum noncytotoxic concentration of 75 μg/mL.	[58]
Fe	Gram-positive bacteria: *S. aureus* Gram-negative bacteria: *P. aeruginosa*, and *E. coli*	Ferric iron reduction	Biomedical	Strong antibacterial effect considering the MIC values of 1.96, 31.25, and 15.75 μg/mL, and MBC values of 1.96, 31.25, and 31.25 μg/mL for *E. coli, P. aeruginosa* and *S. aureus*, respectively.	[59]
	Vírus: Chikungunya virus (CHIKV)	Green synthesis from impregnation into raw Citrus limetta peels	Therapeutic	Antiviral activities of Fe-NPs at concentrations of 0.05 mg/mL, 0.1 mg/mL, and 0.2 mg/mL and an IC_50_ value of 15.52 µg/mL.	[60]
	Fungi: *Fusarium oxysporum, Fusarium tricinctum, Fusarium maniliforme, Rhizoctonia solani*, and *Phythium* sp.	Phyco-synthesis with aqueous extract of the green microalga Chlorella K01	Biomedical	Iron oxide nanoparticles at 1 mg/L inhibited the radial growth of all fungal pathogens tested.	[61]
Fe_3_O_4_	Fungi: *Trichothecium roseum, Cladosporium herbarum, Penicillium chrysogenum, Alternaria alternata* and *Aspergillus niger*	Green synthesis	Biomedical	Antibacterial activity with MIC values of 0.063 mg/mL for *Trichothecium roseum* and *Cladosporium herbarum*, 0.032 mg/mL for *Alternaria alternata* and 0.016 mg/mL for *Penicillium chrysogenum.* and *A.niger*.	[62]
	Vírus: H1N1 influenza	Chemical	Biomedical	A major decrease in viral RNA concentration with the administration of 7.5 pg/mL of iron oxide NPs.	[39]
Ti	Gram-positive bacteria: *S. pneumonia, S. aureus*, and *B. subtilis* Gram-negative bacteria: *S. typhimurium, P. aeruginosa*, and *E. coli* Fungi and Yeast: *C. albicans, C. glabrata, C. guilliermondii*, and *C. krusei*	Green synthesis by *Ziziphora clinopodioides* extract	Cutaneous wound healing; medical, biomedical and pharmaceutical research	Higher antibacterial and antifungal effects than all standard antibiotics and antioxidant effects. The MIC values were 4 mg/mL for *S. typhimurium, E. coli, P. aeruginosa, S. aureus* and *C. albicans* and 2 mg/mL for *S. pneumonia*, *B. subtilis, C. glabrata, C. guilliermondii*, and *C. krusei.* The MBC/MFC the values were 8 mg/mL for *S. typhimurium* and *E. coli*, 4 mg/mL for *P. aeruginosa, S. aureus, S. pneumonia, C. albicans, C. glabrata* and *C. guilliermondii* and 2 mg/mL for *B. subtilis* and *C. krusei.*	[63]
TiO_2_	Gram-negative bacteria: *P. aeruginosa*	Green synthesis with the extract of *Trichoderma citrinoviridae* as a reducing agent	Food, health, and medicine	Inhibit the growth of extremely drug-resistant bacteria at 100 μg/mL and also showed antioxidant potential at this concentration.	[64]
MgO	Gram-negative bacteria: *E. coli* and *P. aeruginosa* Gram-positive bacteria: *Staphylococcus epidermis*, *S. aureus*, *Methicillin-resistant S. aureus (MRSA)* Fungi and Yeast: *C. albicans, C. glabrata*	-	Engineering infection-free medical devices and implants	Bactericidal/fungicidal effects. MIC values of 0.5 mg/mL for *S. epidermis*, 0.7 mg/mL for *S. aureus*, 1.0 mg/mL for *E. coli, P. aeruginosa, MRSA* and *C. glabrata*, and 1.2 mg/mL for *C. albicans*. Moreover, the minimum lethal concentration (MLC) values were 0.7 mg/mL for *S. epidermis* and *S. aureus*, 1.0 mg/mL for *E. coli* and *C. glabrata*, 1.2 mg/mL for *P. aeruginosa* and *C. albicans*, and 1.4 mg/mL for *MRSA*.	[44]
	Gram-positive bacteria: *Streptococcus mutans* and *Streptococcus sobrinus*	-	Biomedical and dental	Antibacterial activity and antibiofilm properties. The MIC and MBC values were determined at 500 μg/mL and 1000 μg/mL, respectively, for both *S. mutans* and *S. sobrinus.*	[65]
	Gram-negative bacteria: *E. coli, P.aeruginosa* and *Aeromonas baumannii* Gram-positive bacteria: *S. pneumoniae* and *MRSA* Fungi and Yeast: *Fusarium solani, A. niger* and *Aspergillus fumigatus*	Green synthesis with marine brown algae *Sargassum wighitii* as the reducing and capping agent	Biological	Potent antimicrobial activities against both human pathogenic bacterial and fungal strains. MIC for MRSA and *P. aeruoginosa* was 256 μg/mL and the MBC was observed at 256 and 1024 μg/mL of MgONPs for each bacteria. Antifungal experiments (using 10–30 μg/mL of MgONPs) showed potent antifungal activity when compared to a positive control (Fluconozole). Also presented cytotoxic activity against lung cancer cell lines in a dose-dependent manner.	[66]
	Virus: Foot-and-mouth disease	-	Therapeutic antiviral agent in foot-and-mouth disease	Virucidal and antiviral activities in the early steps of the replication cycle before virus entry into the cell. It was observed that concentrations higher than 50 μg/mL inactivate the viruses.	[67]
Cu	Gram-negative bacteria: *E. coli* and *P.aeruginosa* Gram-positive bacteria: *S. aureus* and *B. subtilis* Fungi and Yeast: *C. albicans*	Biosynthesis using aqueous *Tilia* extract	Promising in electronic devices: Human cancer therapy	Cytotoxicity against human colon cancer, human hepatic cancer and human breast cancer cells. Relatively high activity against pathogenic bacteria with concentrations ranging from 25 to 200 μg/mL.	[68]
	Gram-negative bacteria: *E. coli, Vibrio harveyi* and *Vibrio parahemolyticus* Gram-positive bacteria: *B. subtilis* and *S. aureus* Fungi and Yeast: *R. solani* and *Sclerotium oryzae*	Green synthesis using Manilkara zapota leaf extract	Antiproliferative, antimicrobial and photocatalytic agent	Antiproliferative effect in breast cancer cell line. Fungicidal activity for *R. solani* and *S. oryzae* at concentrations of 50 μg/mL, 100 μg/mL and 200 μg/mL. As for antibacterial activity *B. subtilis, V. parahaemolyticus, V. harveyi, S. aureus* and *E. coli* presented inhibition at 5 μg/mL concentration of Cu-NPs.	[69]
CuO	Gram-positive bacteria: *S. mutans, Lactobacillus casei*, and *Lactobacillus acidophilus* Fungi and Yeast: *C. albicans, C. krusei*, and *C. glabrata*	-	Dental	High antimicrobial effect against dental caries bacterial agents with MIC_50_ values of CuO-NPs determined at the range of 1–10 μg/mL for *S. mutans*, <1 μg/mL for L. acidophilus, 10 μg/mL for *L. casei* and 1000 μg/mL for *C. albicans, C. krusei*, and *C. glabrata.*	[70]
	Virus: herpes simplex type 1	-	Treatment of oro-labial or genital herpetic lesions	Significant antiviral potency against HSV-1, with the production of ROS through free Cu ions released from the NPs, where the optimal concentration for the antiviral effect was found to be 100 μg/mL.	[71]
Cu_2_O	Gram-negative bacteria: *P. aeruginosa* Gram-positive bacteria: *B. subtilis*	Fabricated in reverse micellar templates by using lipopeptidal biosurfactant as a stabilizing agent	Biocompatible bactericidal and therapeutic	Potent antimicrobial activity through significant ROS generation. The MIC value was found to be 62.5 μg/mL for *B. subtilis* and *P. aeruginosa* microorganisms.	[72]
Au	Gram-negative bacteria: *Klebsiella pneumoniae, S. typhimurium, E. coli* and *P. aeruginosa* Gram-positive bacteria: *Bacillus cereus, B. subtilis, S. aureus* and *Corallium rubrum* Fungi and Yeast: *C. albicans, C. glabrata* and *Cryptococcus neoformans*	Green synthesis using seed extract of mango (*Mangifera indica*)	Therapeutic agents in the biomedical field	Moderate antibacterial, cytotoxic and antioxidant activity. In addition, it exhibited potential cytotoxicity on cancer cell lines. The inhibition of bacteria and fungi corresponded to concentrations of 50 mg/mL of Au-NPs.	[73]
	Gram-negative bacteria: *E. coli* and *P. aeruginosa* Gram-positive bacteria: *B. subtilis* and *Streptococcus* Fungi and Yeast: *Aspergillus* and *Penicillium*	Microwave-assisted method using the leaf extract of *Synedrella nodiflora* as reducing and stabilizing agent	Field of biomedicine and catalysis	Effective antimicrobial activity, significant antioxidant properties and potent catalytic activity. Regarding the antioxidant potential, Au-NPs presented an IC_50_ of 55.40 μg/mL.	[74]
	Virus: Influenza A	Porous Au-NPs were prepared following the surfactant-free emulsion method	M-NPs-based therapy to treat viral infection; Multiplatform for detection of the virus.	Inhibited viral membrane fusion by blocking the viral entry process through conformational deformation of hemagglutinin. The cell viability decreased to less than 60% after 10 min of exposure to 0.2 mg/mL of Au-NPs.	[75]
	Virus: Herpes Simplex	Gallic acid-induced rapid reduction reaction.	Virus chemotherapy.	Prevented viral attachment and penetration. The antiviral efficiency had a EC_50_ value of approximately 32.3 and 38.6 μM for HSV-1 and HSV-2, respectively.	[76]
Ag	Gram-positive bacteria: *S.aureus, P. aeruginosa*	Extracellular by *Pseudomonas hibiscicola*	Drug-resistant strains hospital-acquired infections, treatment and prevention therapy.	Profound synergistic antimicrobial activity against drug-resistant strains of MRSA, extended-spectrum ß lactamases producer (ESBL), vancomycin-resistant *Enterococci* (VRE), and multi-drug resistant (MDR) *P. aeruginosa* with MIC values of 2.5 mg/mL.	[55]
	Gram-negative bacteria: *E. coli* and *P. aeruginosa* Gram-positive bacteria: *B. subtilis* and *Streptococcus* sp. Fungi and Yeast: *Aspergillus* sp. and *Penicillium* sp.	Microwave-assisted method using Leaf extract of *Synedrella nodiflora* as reducing and stabilizing agent	Field of biomedicine and catalysis	Effective antimicrobial activities and significant antioxidant properties. Regarding the antioxidant potential, Ag-NPs presented an IC_50_ of 54.30 μg/mL	[74]
	Gram-negative bacteria: *E. coli O157:H7* Gram-positive bacteria: *Listeria monocytogenes*	Green synthesis with a lyophilized extract from grape and orange wastes	Biomedical	Growth inhibition of bacteria in a dose-dependent manner, with the concentration for inhibition ranging from 20 to 100 μg/mL.	[77]
	Gram-negative bacteria: *E. coli, P. aeruginosa* and *Salmonella typhi* Gram-positive bacteria: *S. aureus* and *Enterococcus faecalis* Fungi and Yeast: *A. niger, C. albicans, Penicillium notatum, Trichoderma viridiae* and *Mucor* sp.	Green synthesis using the leaf extracts of the medicinal plant Tropaeolum majus	Therapeutic drug for microbial infectious disease and other health associated disorders	Antibacterial, antifungal, antioxidant and anticancer properties. MIC values were 3 μg/mL for *S. typhi, S. aureus, E. faecalis* and *C. albicans;* 4 μg/mL for *E. coli* and *A. niger;* 6 μg/mL for *P. aeruginosa* and 8 μg/mL for *P. notatum, T. viridiae* and *Mucor* sp.	[78]
	Fungi: *Verticillium dahliae*	Green synthesis of Ag-NPs using *Melia azedarach* leaf extract	Horticultural applications	Antifungal activity where an application of 60 ppm of Ag-NPs inhibited mycelial growth with significant effects in vivo.	[79]
	Virus: Herpes Simplex types 1 and 2	Modification with tannic acid	Vaginal treatment of genital infection	Affected viral attachment, blocked penetration and cell-to-cell transmission with an administration of 5 ppm of Ag-NPs modified with Tannic Acid.	[80]
	Virus: HAV-10, Herpes Simplex-1 and Coxackie B4 (CoxB4)	Green synthesis by aqueous and hexane extracts of *Lampranthus coccineus* and *Malephora lutea*	Therapeutic and biomedical	Antiviral activity by interaction with herpes simplex thymidine kinase, HAV 3c proteinase and Coxsackievirus B4 3c protease. The IC_50_ of HAV-1, HSV-1 and CoxB4 viruses was 11.71, 36.36 and 12.74 μg/mL, respectively, with *Lampranthus coccineus* hexane nano extract, and 31.38 and 29.04 μg/mL only for HAV-10 and CoxB4 with *Malephora lutea* hexane nano extract.	[81]
	Virus: Severe acute respiratory syndrome coronavirus 2	-	Therapeutic	Potent inhibition of viral entry step via disrupting viral integrity with the administration of concentrations ranging from 1–10 ppm of Ag-NPs.	[82]
	Virus: Respiratory syncytial virus (RSV)	-	Therapeutic	Reduced viral replication and production of pro-inflammatory cytokines in epithelial cell lines and mouse lungs. A reduction of RSV replication was observed in Hep-2 and A549 epithelial cell lines, with an effective dose of 50 μg/mL Ag-NPs. Also, mouse lung tissue incubated with 4 mg/kg Ag-NPs presented a significant reduction in RSV copy numbers.	[83]
	Virus: Human immunodeficiency virus	-	Therapeutic	Exertion of anti-viral activity at an early stage of viral replication and inhibitor of viral entry. The IC_50_ was calculated at approximately 0.44 mg/mL of Ag-NPs and the CC_50_ (cytotoxic concentration) in HeLa cells was determined to be approximately 3.9 mg/mL.	[84]
	Virus: Human Papilloma virus	Green synthesis using *Saccharina japonica* extract	Prevention and treatment of cervical tumors	Cytotoxic effect in the human cervical carcinoma cells, where concentrations between 0.16 and 0.32 mg/mL of Ag-NPs could inhibit HeLa cells growth.	[85]

**Table 3:** Different approaches for the development of metallic antimicrobial surfaces to be applied in healthcare, food industry, and others.

Surface	Coating Method	Metals	Strains	Applications	Main Results	Reference
**Healthcare**						
“Hybrid” nanostructured superhydrophobic PMMA surfaces	Sputtering	Ag, Cu	*Synechococcus* sp. *PCC7942* (cyanobacteria)	Hospital, domestic and public surfaces	Metal-sputtered superhydrophobic surfaces able to promote bacterial repulsion and killing efficacy (due to the Ag and Cu ions).	[4,95]
Polyester surfaces (PES)	Sputtering	Cu	*C. albicans* and *C. glabrata*	Hospital surfaces	Cu-PES displayed fungicidal activity against *C. albicans* and *C. glabrata* within 60 min.	[96]
Polydopamine-coated Ti implants	Spin/spray coating	Ag^+^, Cu^2+^, Sr^2+^, Zn^2+^	*E. coli* and *S. aureus*	Dental and orthopaedic prostheses for implants	All ion coatings showed antibacterial activity, reducing the viability of the tested species by over 85% after 3 h of contact.	[105]
Laser textured Cu surfaces (LT-Cu)	- (no coating)	Cu	MRSA, *P. aeruginosa*, *S. aureus* and *E. coli*	Biomedical surfaces (e.g., hospital handrails and doorknobs)	LT-Cu eradicated *P. aeruginosa* and MRSA, *E. coli* and *S. aureus* in 40, 90, 60 and 120 min, respectively.	[1]
Cu and stainless steel surfaces	- (no coating)	Cu	Influenza A	Surfaces for schools and health care units	Cu inactivated 75% of Influenza A just 1 h, whereas, after 6 h, Cu presented >99.9% viral inactivation.	[99]
**Food industry and Other applications**						
Silicon substrates	Sputtering	Ag, Ti, Cu, Fe, Mo, Zn	*L. monocytogenes, E. coli* and *S. aureus*	Food industries	Cu killed 99% of all the three strains of bacteria, followed by Ag which killed 36, 99 and 34% of *S. aureus, E. coli* and *L. monocytogenes*, respectively. Zn also significantly decreased cell viability of the three strains, Mo and Fe were only effective in killing *S. aureus* and *L. monocytogenes*, whilst Ti was only able to kill *S. aureus*.	[97]
Wide range of Cu-containing alloys (Cu, brasses, bronzes, Cu Nis and Cu-Ni-Zn alloys)	- (no coating)	Cu, Zn, Sn, Ni, Al, Mn, Fe, Cr, P, Si, Ti, Mg (in the alloys)	*E. coli* O157:H7	Food industries and domestic work surfaces	19 out of 21 tested alloys eradicated *E. coli* O157 between 1 to 6 h of contact. Plus, a correlation between Cu content and decreased *E. coli* O157 survival time was found in the bronzes.	[101]
Wide range of Cu-containing alloys (Cus, brasses and other alloys)	- (no coating)	Cu, Zn, Ni, Sn, Fe, Cr, Mn (in the alloys)	HuCoV-229E	Public surfaces	Cu, brasses with Cu >70% and Cu-Ni alloys with Cu >90% had the best virucidal activity, eradicating HuCoV-229E in 20–60 min. Cu ions and ROS formation were responsible for inactivating the virus.	[100]
Stainless steel touch surfaces	Cold-spray coating	Cu	SARS-CoV-2 (COVID-19)	Touch surfaces (e.g., stainless-steel door push plates)	Cu inactivated 96% of the virus in the first 2 h of contact and nearly eradicated it after 5 h presenting an inactivation of 99.2%.	[102]
In vitro test (microplates)	- (no coating)	Ga^3+^	*Aspergillus* spp. and *Candida* spp.	Antifungal therapy	Ga displayed potent antifungal potential.	[103]

**Table 4:** Synergistic interactions between different types of M-NPs and drugs.

NPs	Type of Drug	Strains	Mechanism of interaction	Main Results	Reference
	**Antibacterial**				
Ag	tetracycline, neomycin	*S. typhimurium DT104.*	The complex formed between drugs and Nps might be produced due to interactions established between the positively charged Ag-NPs and the large amount of OH groups found in the composition of both antibiotics, which provides them with a negative charge. Additionally, Tetracycline and Neomycin can bind to the bacteria’s membrane proteins.	Tetracycline alone in concentration ranges of 0.01–1.25 μg/mL does not inhibit bacterial growth, neither neomycin in ranges of 0–9.6 μg/mL. Ag-NPs alone at 5 μg/mL can cause up to 30% of inhibition after 2 h of exposure. Tetracycline combined with Ag-NPs inhibit bacterial growth by 85% with an IC_50_ of 0.07 μg/mL after 2 h of exposure. Neomycin combined with Ag-NPs presents an IC_50_ of 0.43 μg/mL after 2 h of exposure.	[112]
	streptomycin, amikacin, kanamycin, vancomycin, tetracycline, ampicillin, cefepime, amoxicillin, cefetaxime	*S. epidermidis, Serratia marcescens, E. coli, S. typhimurium, K. pneumonia, B. cereus, S. aureus* and *B. subtilis.*	Antibiotics and NPs were used individually and not complexed.	The studied bacteria were found to be inhibited in the presence of the AgNPs and antibiotics combination, which otherwise showed a resistant pattern in the presence of antibiotics (vancomycin, cefetaxime, ampicillin, kanamycin, amikacin, cefepime) alone.	[116]
	bacitracin, kanamycin, gentamicin, streptomycin, erythromycin, chloramphenicol (Ch)	*B. subtilis, E. coli, S.aureus* and *K. pneumoniae.*	Ag-NPs and antibiotic conjugates can be obtained through electrostatic interactions. It is also possible to have hydrophobic interactions as well as covalent bonds between the NPs and sulfhydryl groups (-SH) present on the antibiotics.	For all the bacteria strains, an overall percentage of synergistic bacterial effect between Ag-NPs and antibiotics was observed with 16, 11.5, 10, 87, 9.4 and 9.7% for kanamycin, gentamicin, streptomycin, bacitracin, ch and erythromycin, respectively.	[117]
	chloramphenicol	*B. cereus, B. subtilis, S. aureus, C. rubrum, E. coli, P. areuginosa, S.a typhimurium* and *K. pneumoniae.*	The bonding reaction between the antibiotics and AgNPs could occur due to the chelation process.	The MIC values ranged from 0.312–2.5 and 1.25–2.5 mg/mL and the MBC values ranged from 2.5–>10 and 5–10 mg/mL for Ag-NPs and Ch, respectively. However, when combined, the MIC and MBC values decreased for 0.078–0.625 (Ag) and 1.25–10 mg/mL (Ch), respectively.	[118]
Au	cefotaxime and ciprofloxacin	*S. typhimurium, S. typhi and Salmonella enteritidis.*	Antibiotics and Au-NPs were not complexed. However, the mutual delivery of both species enabled a combined effect of ROS accumulation from the antibiotics effect and membrane disruption, inducing apoptosis due to Au-NPs presence.	*S. typhimurium*: the MIC value for Au-NPs, cefotaxime and ciprofloxacin alone was 2.5 μg/mL for all. When combined the MIC values were 0.65 μg/mL for Au-NPs and cefotaxime and 0.32 μg/mL for Au-NPs and ciprofloxacin. *S. typhi*: the MIC values were 5 μg/mL for Au-NPs and cefotaxime alone and 2.5 μg/mL for ciproflaxin alone. When combined the MIC values were 0.65 μg/mL for Au-NPs and cefotaxime and 1.3 μg/mL for Au-NPs and ciprofloxacin.	[119]
Cu	benzalkonium chloride (BAC)	*E. coli ATCC 25922, S. aureus MRSA 33591* and *S. aureus MRSA 25923.*	Cu-NPs were prepared by electrochemical synthesis and stabilized by sacrificial anode electrolysis method with benzalkonium chloride, forming core-shell Cu-NPs with BAC as a capping agent.	The obtained results showed an uncountable number of colony-forming units (CFU) for Cu^2+^ salt (CuCl_2_), Cu-NPs stabilized with butyl-ammonium perchlorate and tetra-butyl-ammonium perchlorateand; 230 CFUs for BAC alone and 0 CFUs for Cu-NPs combined with BAC. Also, the MIC values for Cu-NPs and BAC combinations are 12.5, <1 and 3.125 μg/mL for *E. coli ATCC 25922, S. aureus MRSA 33591* and *S. aureus MRSA 25923*, respectively.	[120]
	ampicillin, amoxicillin, gentamicin and ciprofloxacilin	*E. coli, S. typhi, Micrococcus luteus* and *S. mutans*	The metallic Cu is known to react with active groups, like amido and hydroxyl, which are present in antibiotic molecules, leading to a synergism between Cu-NPs and antibiotics.	The synergism between Cu-NPs and antibiotics was proved by an increase in the inhibition zone, when both species are combined, presenting 29.06, 7.6, 7.35 and 25.07% of the synergistic activity with ampicilin, amoxicillin, gentamicin and ciprofloxacin, respectively.	[121]
ZnO	glutamic acid, thiosemicarbazide and ciprofloxacin	*S. aureus* isolates and standard *ATCC 25923.*	ZnO-NPs were functionalized with glutamic acid and conjugated with thiosemicarbazide, which can react as a chelating ligand. In this case, ZnO-NPs and ciprofloxacin did not form a complex, but instead, functionalized ZnO-NPs damaged the bacterial membrane and removed essential metal ions present on the cell surface by chelation, disrupting the membrane permeability, and allowing ciprofloxacin to enter the bacterial cell.	When administrated alone, ciprofloxacin and ZnO-NPs presented an average inhibition zone significantly lower than when administrated together.	[122]
	octadecanethiol (ODT)	*S. aureus* and *E. coli*.	ZnO-NPs and ODT were not complexed. Possibly, ODT molecules facilitated the dispersion of ZnO-NPs at the fabric’s surface, preventing the formation of agglomerations.	*S. aureus*: For the ODT and ZnO-NPs alone, the CFU/m^2^ count was 312000 and 4440, respectively. Whereas for the combination of ODT plus ZnO-NPs, the CFU/m^2^ count was 600. *E. coli*: For the ODT and ZnO-NPs alone, the CFU/m^2^ count was 49800 and 1800, respectively. Whereas for the combination of ODT plus ZnO-NPs, the CFU/m^2^ count was 48. The combination of ZnO-NPs and the surfactant ODT significantly diminished the bacterial adhesion.	[123]
MgO	nisin	*E. coli* and *Salmonella stanley.*	The synergistic effect of MgO-NPs and nisin is not clear. However, nisin treatment might have been responsible for the rupture of large pores in the bacterial cell membrane, allowing the entering of MgO-NPs into the cell, which can produce ROS and damage the bacterial DNA.	*E. coli*: the logarithmic CFU/mL levels were approximately 3 and 4 when 25 mg/mL of nisin and 4 mg/mL of MgO-NPs, respectively, were administrated alone. Whereas, when they were administrated together, the logarithmic CFU/mL level came down to approximately 1. *S.stanley*: the logarithmic CFU/mL levels were approximately 3 and 4 when 25 mg/mL of nisin and 4 mg/mL of MgO-NPs, respectively, when administrated alone. Whereas, when they were administrated together, the logarithmic CFU/mL level came down to approximately below 3.	[124]
	**Antifungal**				
Ag	Amphotericin B (AmB)	*C. albicans, C. glabrata* and *C. neoformans.*	The bonding reaction between the antifungal and AgNPs might be chelation, which altered the membrane permeability and morphology.	When alone, the MIC values were 2.5 and 5 mg/mL for Ag-NPs and AmB, respectively, whereas the MFC values were 10 and >10 mg/mL for Ag-NPs and AmB, respectively. However, when they were combined, the MIC and MFC values decreased to 0.156 and 2.5 mg/mL, respectively.	[118]
	tebuconazole, propineb, fludioxonil	*Bipolaria maydis*	Antifungals and NPs were used individually and not complexed.	The synergism between antifungal compounds and Ag-NPs was measured by the percentage of inhibition towards *B. maydis* ranging from 46–58% for the Ag-NPs alone 48.28, 47.27 and 52.13% for the fungicides Tebuconazole, Propineb, Fludioxonil, respectively, and with values of 73.08, 64.56 and 62.13% for the combination of Ag-NPs with tebuconazole, propineb, fludioxonil, respectively, therefore proving a synergistic interaction between the NPs and the fungicides.	[125]
	zineb	*Neoscytalidium dimidiatum*.	Chitosan was used to functionalized with Ag-NPs and improve the NPs stability, then this compound was combined with zineb and meant to improve fungicidal effect.	To prove the synergistic effects between Ag-NPs and zineb, the inhibition zones were measured and were high when in combination.	[126]
	**Antiviral**				
Ag	oseltamivir (OTV)	H1N1 influenza virus.	Ag-NPs and OTV were synthesized together, being the OTV on the surface of the Ag-NPs. It is believed that Ag-NPs can facilitate OTV’s entry into the cell to exert its antiviral action by reducing ROS and p53 levels.	For the infection growth in MDCK cells, the virus alone diminished cell viability to below 40%, virus+OTV presented viability below 60% and the virus+Ag-NPs close to 70%. Virus+Ag-NPs+OTV reached almost 100% of cell viability. Similar results were obtained for the mitochondrial membrane potential, proving the synergism between Ag-NPs and the antiviral OTV.	[127]
Ag & Au	FluPep	Influenza type-A virus.	Ag-NPs and Au-NPs were used as probes to deliver FluPep onto the cell. The system was based on a shell of NPs around the peptideFluPep.	Ag-NPs and Au-NPs in combination with the peptide provide better antiviral activity than FluPep alone. FluPep alone had IC_50_ values ranging from 1–5 nM, whilst in combination with Au-NPs and Ag-NPs, the value of IC_50_ decreased to 0.015 nM, proving a positive synergistic activity between the antiviral peptide and the NPs.	[128]

## Data Availability

Not applicable.
